# The Effect of Build Angle and Artificial Aging on the Accuracy of SLA- and DLP-Printed Occlusal Devices

**DOI:** 10.3390/polym16121714

**Published:** 2024-06-16

**Authors:** Bardia Saadat Sarmadi, Franziska Schmidt, Florian Beuer, Dilan Seda Metin, Philipp Simeon, Robert Nicic, Alexey Unkovskiy

**Affiliations:** 1 Department of Prosthodontics, Geriatric Dentistry and Craniomandibular Disorders, Charité—Universitätsmedizin Berlin, Aßmannshauser Street 4-6, 14197 Berlin, Germany; bardia.saadat-sarmadi@charite.de (B.S.S.); franziska.schmidt2@charite.de (F.S.); florian.beuer@charite.de (F.B.); dilan-seda.metin@charite.de (D.S.M.); philipp.simeon@charite.de (P.S.); robert.nicic@charite.de (R.N.); 2 Department of Dental Surgery, Sechenov First Moscow State Medical University, Bolshaya Pirogovskaya Street, 19c1, Moscow 119146, Russia

**Keywords:** DLP, SLA, 3D printing, additive manufacturing, occlusal splint, accuracy, artificial aging

## Abstract

The aim of this study is to investigate the influence of printing material, build angle, and artificial aging on the accuracy of SLA- and DLP-printed occlusal devices in comparison to each other and to subtractively manufactured devices. A total of 192 occlusal devices were manufactured by one SLA-printing and two DLP-printing methods in 5 different build angles as well as milling. The specimens were scanned and superimposed to their initial CAD data and each other to obtain trueness and precision data values. A second series of scans were performed after the specimens underwent an artificial aging simulation by thermocycling. Again, trueness and precision were investigated, and pre- and post-aging values were compared. A statistically significant influence was found for all main effects: manufacturing method, build angle, and thermocycling, confirmed by two-way ANOVA. Regarding trueness, overall tendency indicated that subtractively manufactured splints were more accurate than the 3D-printed, with mean deviation values around ±0.15 mm, followed by the DLP1 group, with ±0.25 mm at 0 degree build angle. Within the additive manufacturing methods, DLP splints had significantly higher trueness for all build angles compared to SLA, which had the highest mean deviation values, with ±0.32 mm being the truest to the original CAD file. Regarding precision, subtractive manufacturing showed better accuracy than additive manufacturing. The artificial aging demonstrated a significant influence on the dimensional accuracy of only SLA-printed splints.

## 1. Introduction

Occlusal splints are a valid treatment option for temporomandibular joint dysfunction (TMD), protecting the teeth from excessive antagonist wear or positioning teeth for aesthetic treatments [1,2,3]. The conventional way to manufacture occlusal splints is vacuum thermoforming or injecting heat curing acrylic resin into a silicone form after premodeling the occlusal device in wax [4,5]. Both have been used for over 50 years [6,7,8] and require time-consuming manufacturing steps [9].

Through digitalization of the medical field and the advent of computer-aided design and computer-aided manufacturing (CAD/CAM), a virtual design and subtractive manufacturing of occlusal devices became feasible [10,11]. A better time efficiency as well as similar or superior mechanical properties have been reported as advantages of the digital workflow in this field compared to an analogue workflow [12,13,14,15,16,17]. However, subtractive manufacturing has also its disadvantages, such as high material wasting and limited manufacturing resolution limited by bur size [18,19].

Since the last decade, it has become possible to fabricate occlusal splints by additive manufacturing (AM) [20]. A common AM technology applied in dentistry is VAT photopolymerization [21,22], where a liquid photosensitive resin is selectively cured in a VAT by light-activated polymerization. Two different technologies can be considered: stereolithography (SLA) and digital light processing (DLP). SLA uses a laser to selectively cure each point of the layer of the printed object. DLP uses a two-dimensional UV light projection, thereby curing the complete layer at once [23]. Due to this, DLP is more time-efficient than SLA. AM, also known as 3D-printing, is more material-efficient compared to subtractive manufacturing (SM), as the material waste is limited to the supporting structures of the build object [23,24]. Printing layerwise implicates that there are small gaps between each layer. Due to weak interlayer adhesion forces, material failure might occur in the direction of these gaps, which may result in varying features of polymer-printed objects related to the number of layers used. Depending on the AM technology 3D-printed objects may show an anisotropic behavior because the build angle on the printing platform influences the number of layers and, therefore, printing time [25,26,27].

Regarding CAD/CAM manufacturing of splints, it has been shown that, in general, SM is more accurate than AM [28,29,30]. Unkovskiy et al. demonstrated the general tendency that printing along the object’s *z*-axis negatively affects the accuracy [31]. Studies by Reymus and Cameron confirmed lower trueness with an increasing build angle [28,32]. Furthermore, Reymus and Wesemann described higher trueness within AM with a horizontal build angle [28,29] and higher precision with a vertical build angle, except for SLA printers which showed higher precision positioning the splints horizontally [28].Comparing other printing resins, this difference in accuracy between DLP and SLA printing has been confirmed with better trueness in SLA printing and comparable precision [33]. A study by Vasques et al. showed that the orientation of the splint results in differences in the clinical fit [34], whereas it does not matter when controlling the fit on a stone model [35].

Another important factor regarding occlusal splints is how they react to aging as the fit or occlusal contacts could be influenced by dimensional changes within the splint material over time. Lutz described a volume loss of AM splints after artificial aging [18]. In addition, it has been shown that AM splints have a lower surface roughness after aging than SM [36]. However, a negative effect of artificial aging on mechanical properties of AM splints materials has also been described [26,37]. A negative effect of aging on accuracy and dimensional stability of other printing resin materials for casts or denture bases has also been described in some studies [38,39].

To the best of our knowledge, such aspects of occlusal splint manufacturing as material efficacy, accuracy between various 3D printing methods, influence of build angle, and splint longevity have not been sufficiently investigated yet in one consistent study that takes aging into consideration and has enough data for trueness and precision of SLA and DLP printing. For these reasons, the aim of the present study was to compare the SLA and DLP methods to the subtractive manufacturing with regards to the best cost–time relation, influence of the build angle on the printing accuracy, and influence of artificial aging. The first null hypothesis of this study was that there would be no differences in accuracy between SM, SLA-, and DLP-manufactured occlusal splints. The second null hypothesis was that the build angle does not affect the accuracy of 3D-printed occlusal splints. The third null hypothesis was that artificial aging does not affect the accuracy of occlusal splints.

## 2. Materials and Methods

### 2.1. Sample Manufacturing

#### 2.1.1. CAD

**Figure 1:**
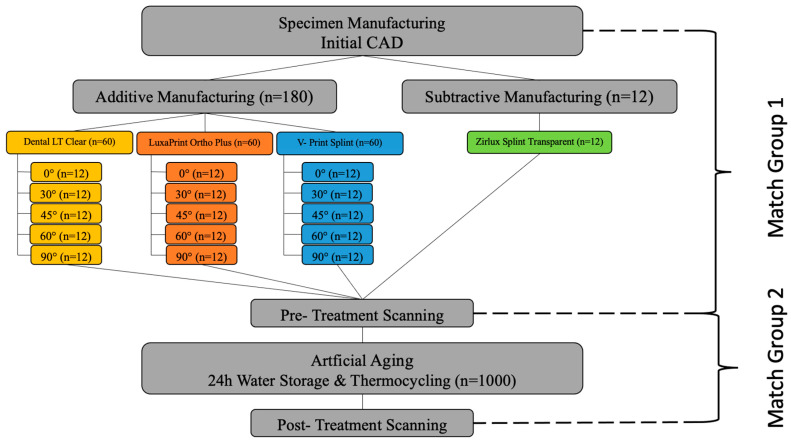
Study flowchart.

A typodont model was uploaded into CAD software Exocad 3.0 (Exocad, Rijeka, Darmstadt, Germany) and an occlusal splint was designed and exported in standard tessellation language STL format. The minimal occlusal thickness was set to 2 mm and the peripheral thickness to 3 mm. This file was selected as the reference CAD data and was manufactured using one SLA, two DLP printers, and a milling machine (Figure 1). Each specimen was printed or milled and post-processed according to the manufacturing instructions. 

#### 2.1.2. Additive Manufacturing

**Figure 2:**
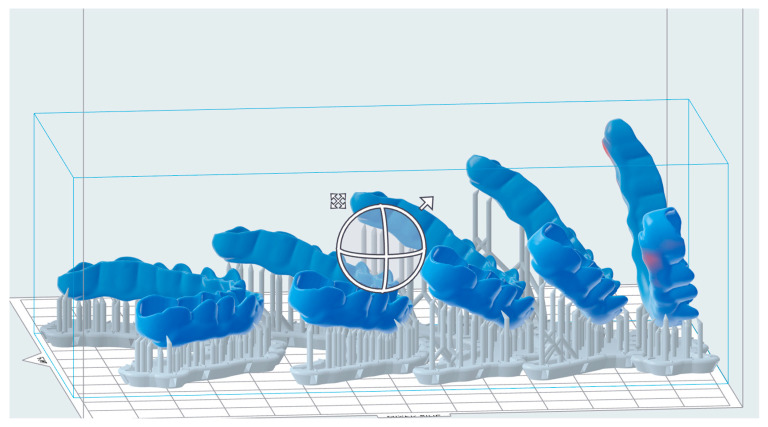
Printing orientation of the splints from left to right: 0°; 30°; 45°; 60°; 90°.

For DLP, the reference file was imported into the slicing software Netfabb Basis 2022.2.2 (Autodesk Inc., San Rafael, CA, USA). The layer thickness was set to 50 µm. The build angle was set by rotating the splints around their *z*-axes by typing the angle into the navigation tool. This way, the build angle was set exactly to 0°, 30°, 45°, 60°, and 90° (Figure 2) for each material and each printer. The supporting structures were added using the integrated “splint” script for each resin. For SLA, it is the automatically generated support script for Dental LT Clear, for DLP1, it is the “splint”, and for DLP2, it is the “V- Print splint thick support bars (bite splint) V01.01”. 

For the SLA group, the file with all five splints, including the supporting structures, was exported in STL format and imported into the integrated slicing software of the SLA 3D printer (Preform, Formlabs, Sommerville, MA, USA). The file was sliced and manufactured with Dental LT Clear material (Formlabs, Sommerville, MA, USA) in the SLA 3D printer (Form 3, Formlabs, Sommerville, MA, USA).

For the two DLP groups the arranged print job was sliced in the Netfabb Basis software (Autodesk Inc, San Rafael, CA, USA). The sliced print job was printed first with LuxaPrint Ortho Plus material (DMG, Hamburg, Germany) in the 3Demax 3D printer (DMG, Hamburg, Germany) and then with V- Print Splint materials (VOCO GmbH, Cuxhaven, Germany) in the Solflex 170 3D printer (VOCO GmbH, Cuxhaven, Germany) (Table 1). The print job was manufactured 12 times for each 3D printer, resulting in 180 splints total.

#### 2.1.3. Subtractive Manufacturing

Prefabricated Zirlux Splint Transparent (ZST) PMMA Blanks were used for the milled splints (Zirlux, Melville, NY, USA). The same CAD data used for additive manufacturing were imported into the nesting software Programill CAM v4 (Ivoclar Vivadent, Schaan, Liechtenstein) and milled using a 5-axis milling machine PrograMill PM7 (Ivoclar Vivadent, Schaan, Liechtenstein) from the refabricated Zirlux Splint Transparent (ZST) PMMA Blanks (Zirlux, Melville, NY, USA). Each blank could be used to manufacture two splints. A total number of 12 splints were milled.

After manufacturing, the splints milled out of one blank and the equal number of printed splints were placed on a scale together with the material that cannot be used after manufacturing to assess the direct material waste comparison.

#### 2.1.4. Post-Processing

The 3D-printed splints were post-processed, including washing and post-curing. 

SLA-manufactured splints (Dental LT Clear) were washed in the Formwash machine (Formlabs, Sommerville, MA, USA) using 99% isopropanol and, after drying, UV cured in the Form Cure machine (Formlabs, Sommerville, MA, USA) at 80 °C. 

For Luxaprint Ortho Plus, the washing was performed using the material specific washing and drying protocol of the 3DeWash machine (DMG, Hamburg, Germany) with 99% isopropanol followed by post-UV curing in the 3 DeCure machine (DMG, Hamburg, Germany). The time and other settings for washing and curing were set by an integrated material-specific script.

For V-Print Splint, the washing was performed in an isopropanol ultrasonic bath for 3 min followed by main cleaning in a fresh isopropanol ultrasonic bath for 2 min. After drying with compressed air, the splints were post-cured using the Otoflash G171 (NK- Optik GmbH, Baierbrunn, Germany) with 2000 flashes and another 2000 flashes after a 2 min cooling time in between (Table 2).

The supporting structures of the 3D-printed splints were removed after washing a curing using a cutting disc and a carbide cutter. For the milled material, the splints were cut out of the blanks using a metal cutter on an electronic handpiece.

### 2.2. Digitization

The manufactured splints were digitized with an industrial scanner (D2000, 3Shape, Copenhagen, Denmark) within 24 h after fabrication to check the accuracy initially after fabrication. Within these 24 h, the specimens were stored in non-UV-transmitting boxes. According to the manufacturer, the scanner is accurate to 5 micrometers. The scanner was calibrated after each experimental group, so the scanning conditions of all groups were the same. “Multi-Line technologie” was used, which means that there were multiple laser lines scanning the object moving on its scanning platform in the scan chamber to provide high scan quality. While obtaining a good image of the intaglio and the outer splint surface, the scanner is not capable of performing a full 360° scan of the object.

### 2.3. Artificial Aging

Each test group was pretreated in distilled water for 24 h before aging began (Figure 2).

Aging was performed by alternately immersing the specimens through a mechanically driven swivel arm into a warm- (55 °C) and a cold-water bath (5 °C) for 30 s each. This cycle was repeated 1000 times. This was intended to represent a clinical wear time of one year where the splint is taken out for 3 meals a day. Hence, 3 times 365 days a year makes an approximal number of 1000 cycles realistic. Within 24 h of the end of this thermocycling protocol, the splints were scanned for the second time.

### 2.4. Evaluation of the Accuracy

**Figure 3:**
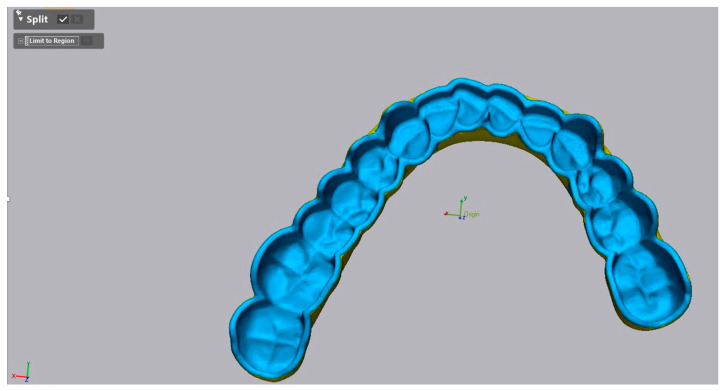
The segmentation function in Geomagic software and the marked intaglio surface of the CAD file used to match with the study specimens.

**Figure 4:**
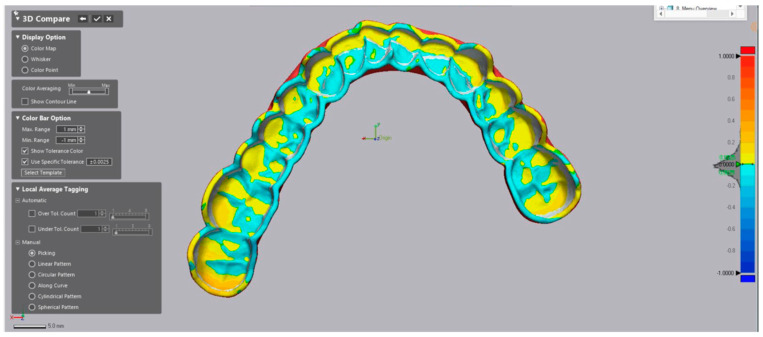
Showing the superimposition of a splint of the DLP2 group after the described alignment protocol of transform alignment and best fit alignment comparing the intaglio surface of the splint.

The accuracy investigation encompassed trueness and precision analysis as per ISO Standard [43]. For analysis of trueness, the obtained scans pre-artificial aging were aligned to the reference CAD data (match group 2) in the metrology software (Geomagic Control X 2022, 3D Systems, Luxembourg, Luxembourg) (Figure 3 and Figure 4). First, the intaglio surface of the reference CAD data was segmented using the “Split” tool, so only the intaglio surface could be matched (Figure 3).

The initial matching was performed using the “Transform Alignment” algorithm which means manually determining three common overlay points of the intaglio surface. The final alignment was performed using the “Best-Fit” algorithm. For analysis of precision, the obtained STL files of each printed denture were matched to each other within each group.

For the quantitative analyses, the dimensional differences were recorded in the root mean square (RMS) error. The RMS was calculated by means of the following formula:

$$\mathrm{RMS}=\sqrt{\frac{\sum_{i=1}^{n}{\left(x_{R,i}-x_{T,i}\right)}^{2}}{n}}$$

where n describes the number of measured points, x_R,i_ stands for the reference data, and x_T,i_ refers to the measurement point of the scanned data.

For the qualitative analysis of trueness and precision, a heat map was generated for each dataset. The range of the maximum and minimum values was set to 1 mm. The tolerance level was set to ±0.025 mm as it represents the maximum *z*-axis resolution of the used AM methods of 0.05 mm.

To assess the effect of artificial aging, the scans after artificial aging were matched to the scan before the artificial aging (match group 2) (Figure 4). A total sum of 2688 matches were made to obtain the data.

### 2.5. Statistical Analyses

All gathered data were statistically analyzed in JMP 14 (SAS Corp., Heidelberg, Ger-many). First, the data were tested for normality by goodness of fit with the Shapiro–Wilk test. For normally distributed data, the statistical difference was analyzed by using two-way analysis of variance (ANOVA) with printing techniques and orientations as two independent factors. Tukey test was further performed for multiple comparisons analysis. The threshold for significance was defined as a *p*-value less than 0.05.

## 3. Results

### 3.1. Quantitative Analyses

The Shapiro–Wilk test revealed a normal distribution of the gathered data. A statistically significant influence was found for all main effects: manufacturing method (F (1, 24) = 164.7, *p* < 0.0001); build angle (F (2, 24) = 16.39, *p* < 0.0001); and thermocycling (F (1, 24) = 164.7, *p* < 0.0001), confirmed by two-way ANOVA.

**Figure 5:**
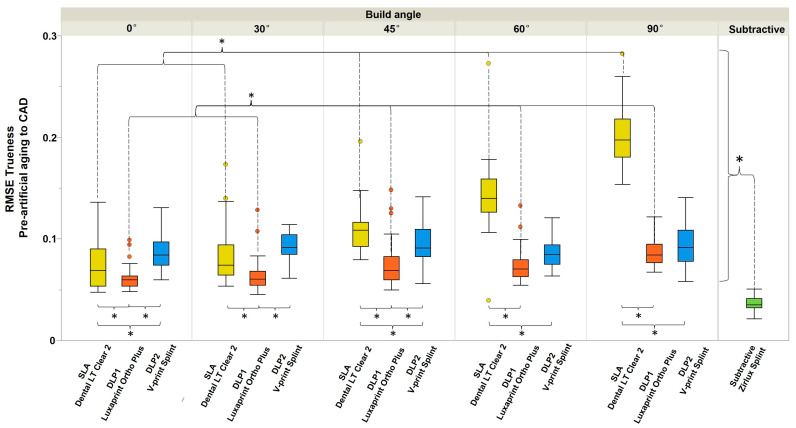
The RMSE (trueness) of four study groups according to manufacturing method and build angle before artificial aging. The asterisks indicate statistically significant difference. SLA and DLP1 0° and 30° demonstrated significantly higher trueness than other build angles. No differences were observed in the DLP2 group.

Regarding the trueness, the overall tendency indicated that subtractively manufactured splints were more accurate than the 3D-printed (*p* < 0.001) (Figure 5). Between the additive manufacturing methods, DLP1 and DLP2 splints had significantly higher trueness for all build angles compared to SLA (*p* < 0.001), confirmed by Tukey’s multiple comparisons tests. The 0° and 30° build angles showed significantly higher trueness for SLA and DLP1 (Luxaprint Ortho Plus, DMG) but not for DLP2 (V- Print Splint, VOCO), compared to other build angles (*p* < 0.001). The increase in the build angle demonstrated the negative effect on the trueness of the SLA and DLP1 (Luxaprint Ortho Plus, DMG) groups and had no influence on the DLP2 group (V- Print Splint, VOCO).

**Figure 6:**
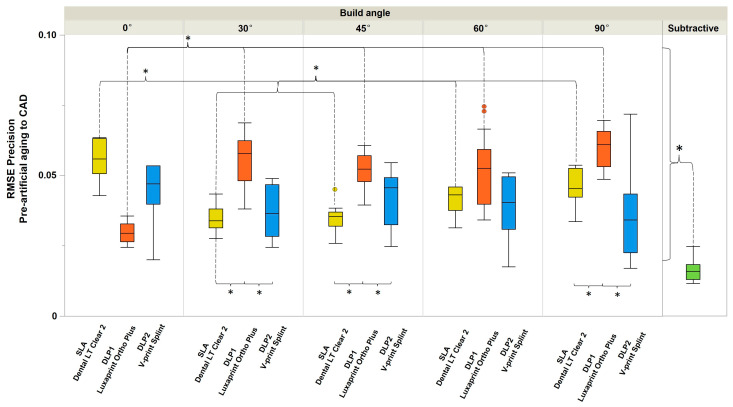
The RMSE (precision) of four study groups according to manufacturing method and build angle before artificial aging. The asterisks indicate statistically significant difference. SLA 30° and 45° demonstrated significantly higher precision than other build angles. DLP1 0° demonstrated significantly higher precision than other build angles. No differences were observed in the DLP2 group.

Regarding the precision, overall subtractive manufacturing showed better accuracy than additively manufacturing (Figure 6). For the DLP1 (Luxaprint Ortho Plus, DMG) group, the 0° build angle showed significantly higher precision than other build angles, whereas for SLA, the 0° build angle showed significantly lower precision. The same was not seen for the DLP2 (V- Print Splint, VOCO) group. For 30°, 45°, and 90° build angles, the SLA and DLP2 (V- Print Splint, VOCO) groups showed significantly higher precision compared to the DLP1 (Luxaprint Ortho Plus, DMG) group.

Except for the 30° build angle for the DLP1 (Luxaprint Ortho Plus, DMG) group and 0° for SLA, a negative effect of an increasing build angle on precision can be seen within those groups but not for the DLP2 (V- Print Splint, VOCO) group.

**Figure 7:**
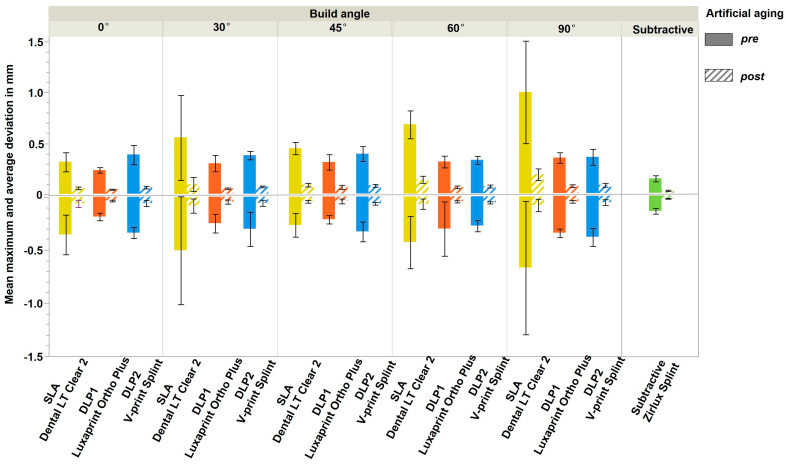
The mean maximum (solid) and mean average (pattern) deviations.

With regards to the mean maximum and mean average deviation, there was a slight domination of positive deviations over the negative (Figure 7). Subtractive manufacturing, again, demonstrated the best trueness, compared to 3D-printed splints (*p* < 0.001).

**Figure 8:**
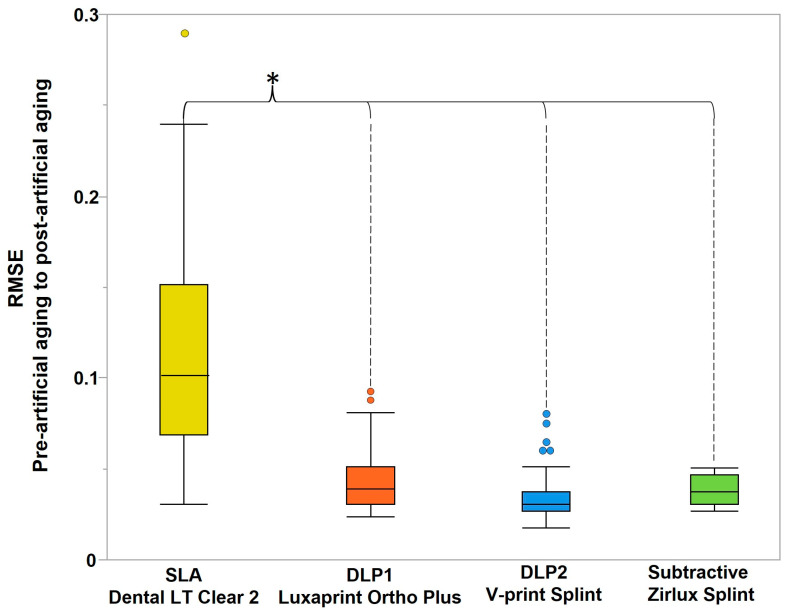
The RMSE (trueness) values between the pre-artificial aging and post-artificial aging comparison, indicating the SLA group to be significantly (*) the most susceptible to thermocycling.

The artificial aging demonstrated a significant influence on the dimensional accuracy of only SLA-printed splints in comparison to DLP and subtractive (Figure 8).

### 3.2. Qualitative Analyses

**Figure 9:**
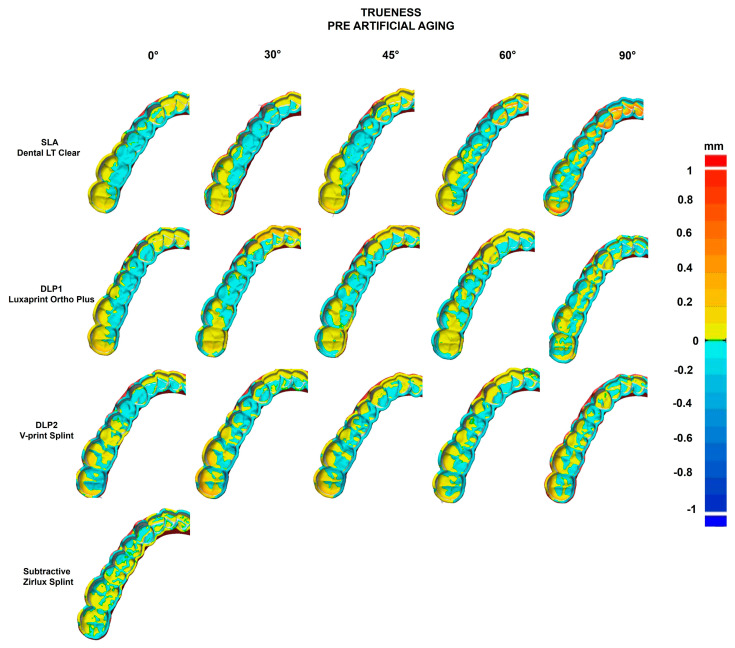
Heat map showing the trueness of each manufacturing method before artificial aging: SLA, DLP1, DLP2, and subtractive. The minimal and maximal range was set to ±1 mm, and the tolerance was set to ±0.005 mm. Positive values (yellow, red) indicate a convex, negative values—a concave.

The trueness heat map before artificial aging (Figure 9) demonstrated greater positive deviations for the SLA group by 90° build angle (yellow to red). Most positive deviations were concentrated in the posterior area in all additive manufactured splints, whereas the subtractive splint demonstrated a rather even distribution of slight positive deviations.

**Figure 10:**
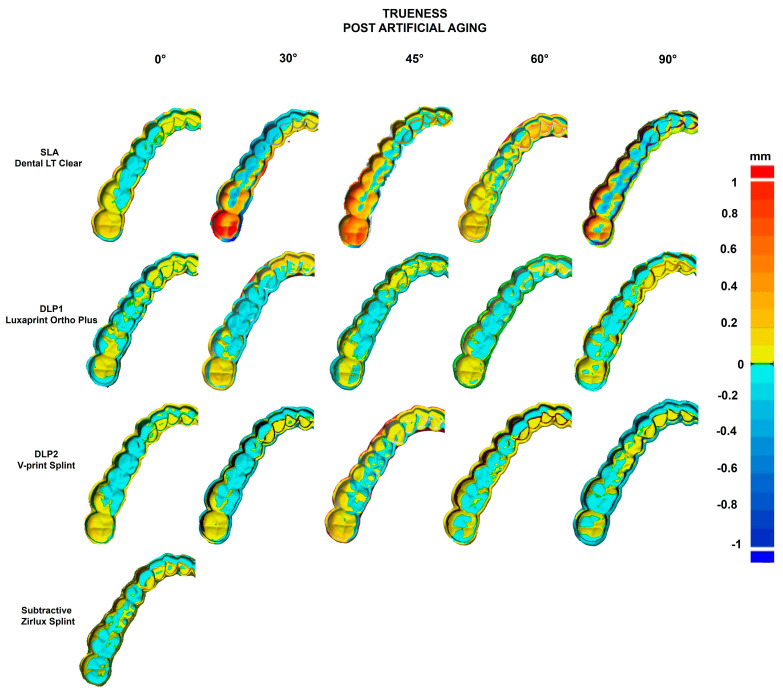
Heat map showing the trueness of each manufacturing method after artificial aging: SLA, DLP1, DLP2, and subtractive. The minimal and maximal range was set to ±1 mm, and the tolerance was set to ±0.005 mm. Positive values (yellow, red) indicate convexes, negative values—the concaves.

The trueness heat map after artificial aging (Figure 10) demonstrated much greater positive deviations for the SLA group for all build angles (yellow to red). With regards to the DLP groups, the most positive deviations after artificial aging were concentrated in the posterior area in all additive manufactured splints, whereas the subtractive splint demonstrated a rather even distribution of slight positive deviations.

### 3.3. Material Usage Analysis

**Figure 11:**
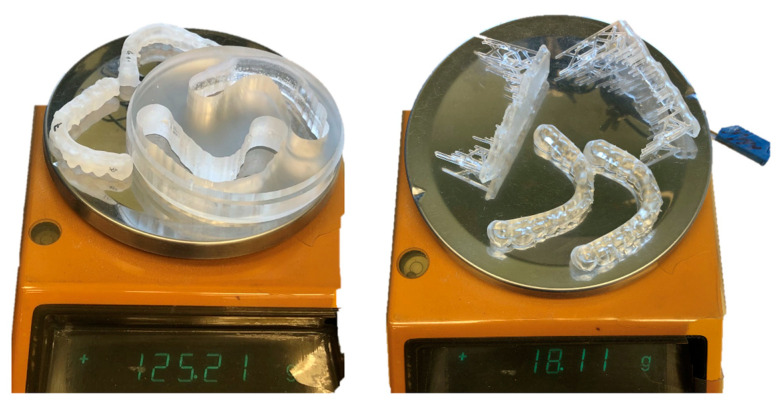
The difference in material usage between milling two splints and printing two splints (SLA, 90° build angle). A much higher waste of material can be seen in milled splints.

Figure 11 shows a much higher amount of material used for milling two splints in comparison to printing two splints. The material used for printing is only 14.46% of the material used for milling.

## 4. Discussion

### 4.1. Results

Manufacturing method, build angle, and artificial aging influenced the accuracy of occlusal splints. Therefore, all three null hypotheses must be rejected.

Accuracy was defined by trueness and precision as described in ISO 5725. Trueness represents the closeness of agreement between test results (scans of manufactured splints) and the true reference (original CAD data) and precision stands for closeness of agreement between test results (scans of manufactured splints compared to each other) [43].

First, the subtractively manufactured splints appeared to be more accurate compared to additively manufactured splints. This outcome confirms previous studies [28,29,30]. Furthermore, within the AM group, the printing method, the resin, and the build angle showed significant influence on trueness and precision. SLA demonstrated significantly lower trueness than DLP. This outcome coincides with a previous study of Marcel et al. [28]. The reason for that could be explained by the different light/laser distribution. While SLA polymerizes each point of the object, DLP printers polymerize one whole layer at a time. This leads to more light exposure for each cured point in SLA, which may result in unwanted dimensional changes due to overcuring. However, this result contradicts other studies, showing better trueness for SLA-printed complete denture bases [33,44]. The difference between the DLP1 and DLP2 groups is an indication that the material itself plays an important role [45,46]. Different material components like various functional groups can lead to different mechanical properties, as shown in other studies [46,47,48].

The present study demonstrated that build angle affects the trueness and precision of printed splints. The strongest influence of build angle is observed in SLA regarding the trueness, where an increasing build angle has a negative effect on the trueness and precision. As for DLP, a negative effect of increasing the build angle was observed for the DLP1 group, with the highest trueness and precision in 0°, but not for the DLP2 group, which did not show any significant differences in trueness or in precision. Cameron et al. also found differently behaving trueness values for two DLP printers in the recent study [32].

Decreasing accuracy values with increasing build angle have been described in studies before, showing that build angles between 0° and 45° show significantly better accuracy than 90° [28,49]. A similar behavior is described for other resins, i.e., surgical template resins [50,51] or complete denture base resins [52], which affirms the assumption of a direct influence of build angle on the accuracy of additively manufactured devices. The analysis of mean maximum and mean average deviations over the whole splint geometry indicated a dominance of positive deviations. This finding may be attributed to insufficient removal or an overcuring of excess on the already polymerized parts during the printing and post-processing.

The artificial aging had a selective negative effect on 3D-printed splints. Thus, the SLA splints were significantly more susceptible to thermocycling compared to DLP1 and DLP2. This finding is confirmed by the results of a study by Revilla Leon et al. [49]. In general, there are several studies indicating an overall negative affect of aging on printable resins [14,18,37,39,53,54], and also one recent study that indicates that although there are negative effects on the mechanical properties, those are not significant [36]. Some other studies have proven the negative effect of aging on other 3D-printed objects, like complete denture bases and models [39,55]. The material composition is, again, a factor that plays a role in the effect of aging on the accuracy, as it has been shown that methacrylate monomer resins are more affected by aging, which is in line with the outcome of this study [56].

### 4.2. Clinical Significance

The differences found in RSME value between SM, SLA, and DLP should be carefully considered in a clinical setting. To better understand the clinical implications of the variations in RMSE values between SLA and DLP methods, we conducted a further evaluation, focusing on the average difference in millimeters. Overall, there is a slight dominance of positive deviations.

SM shows the best trueness values with ±0.15 mm, and DLP1 (Luxaprint Ortho Plus, DMG) with ±0.25 mm. It has been shown that printing in a 0° build angle takes the least amount of time [50], while a 90° build angle lets the producer print the most splints at a time (Figure 1). According to a clinical study from 2018 using the same SLA material, splints with 0° build angle fit very well [34], even though they had a mean average deviation of ±0.32 mm. DLP splints at 90° build angle are within these deviations and, hence, would fit in the patient’s mouth, and a 90° build angle could be used in the daily practice to print more splints and use the least amount of material. It is important to note that Vasques et al. had a very small sample size of *n* = 2, which cannot be considered representative. Clinical studies with a higher sample size are needed to really evaluate the fit of vertically printed splints.

Looking at the heat maps, the positive deviations are mostly in the posterior area of the dental arch (Figure 9 and Figure 10), especially for 90° build angle SLA splints, whereas milled splints showed an even distribution. That could lead to wrong transfer of the bite relation and prevent the desired treatment outcome or lead to more time being spent correcting the bite. Grymak et al. also described higher surface roughness and the need for a greater polishing effort for 90° build angle compared to lower build angles in SLA and DLP [15]. Therefore, 90° build angle printed splints seem unsuitable for clinical use rather than 0° and 30° build angle splints.

Recently, Herpel et al. conducted an in vivo pilot study comparing printed and milled splints in a three-month timeframe. They printed a flexible resin in 0° build angle using a DLP printer and tested the fit, wear behavior, and patient satisfaction in comparison to milled splints [57]. Their results show that while milled splints showed less wear abrasion, all the printed splints had a sufficient fit, survived the three months’ time, and had an even satisfaction grade. Printed splints showed a higher crack formation in the posterior region, which can be explained by higher deviations found in those areas found in this study. That could also lead to a higher fracture risk over a longer wear period.

Taking into consideration that the material waste is much less in printing (Figure 11), the results of our study implicate that the use of DLP-printed occlusal splints is a viable clinical treatment option. However, more clinical studies with more materials, i.e., soft splint materials and different build angles, should be conducted to further assess the optimal printing modifications for clinical use. Moreover, different post-processing procedures should be included as it has been shown that, e.g., post-curing in a nitrogen gas atmosphere enhances mechanical properties and reduces aging effects on occlusal splint materials [58].

### 4.3. Limitations

**Figure 12:**
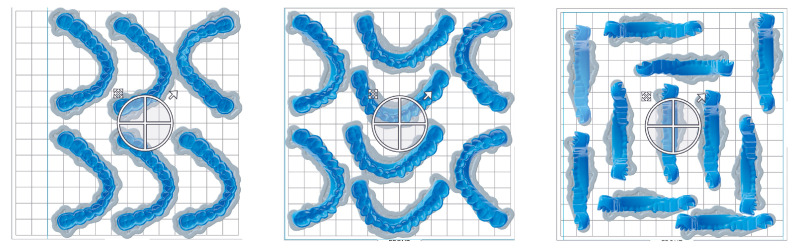
Different number of splints able to be printed at a time in slicing software PreForm 3.14.0 (Formlabs, Sommerville, MA, USA). **Left**: with 0° build angle, 6 splints can be printed at a time; in the **middle**: 8 splints using 30° build angle; on the **right**: 11 splints with 90° build angle.

The in vitro setting of the study can be seen as a limitation due to its limited applicability to clinical reality. However, establishing this kind of study in vivo would be very difficult because of many factors, which could influence the outcome. Especially, the number of printing parameters investigated in this study would mean that the number of patients taking part must be enormously high regarding the possibility of patients dropping out or that they cannot be included due to incompliance. The confirmation of that challenge can be seen in the study of Herpel et al.—the only in vivo study with 23 patients receiving printed splints [57]. Only 0° DLP splints were printed, which means that different build angles, material settings, or AM methods were not taken into consideration. Following simple multiplication, a study that contains all the parameters of this study must onboard approximately 300 patients, considering also the dropout rate. The future in vivo work could focus on comparing different DLP materials to each other regarding accuracy, aging, and wear behavior with different post-processing procedures, i.e., post-curing in nitrogen gas atmosphere and different build angles, which directly influence the number of splints printed at a time, as that is an important clinical factor. A possible build angle split could be 0°, 30°, 90° as shown in Figure 12.

Another limitation of the study is the lack of a standard aging protocol, especially regarding the number of cycles. Aging was performed through thermocycling the splints for 1000 cycles in a cold and warm water bath (5 °C/55 °C) with a 30 s dwell time. In 1994, the International Organization for Standardization suggested that 500 cycles are enough to adequately represent aging of biomaterials in their ISO TR 11405 [59]. Nowadays, that standard is not widely accepted anymore. Although Gale and Darvell suggested a number of 10,000 cycles for an approximate use of one year [60], that suggestion is not backed by scientific evidence and, thus, most authors do not acknowledge it as a real standard mostly using less cycles. Therefore, every aging protocol is based on the logical thoughts of the author [61]. As the wear time of occlusal splints can differ from a few days to over a year, an exact test duration for artificial aging is hard to determine [62]. Therefore, to use an average timeframe, a one-year duration was selected.

More studies with a larger range of cycle numbers are needed to further investigate the influence of artificial aging.

The accuracy of the scanner used might also be a limitation; however, 5 µm, according to DIN ISO Standard (D2000, 3Shape, Copenhagen, Denmark) [63,64], is comparable to studies investigating a similar topic that use the same or a comparable laboratory scanner [29,65,66].

## 5. Conclusions

Within the limitations of the present study, the following conclusions can be drawn:

1. Subtractive manufacturing shows better trueness and precision than additive manufacturing.
2. DLP shows better trueness than SLA, whereby the 0° and 30°are significantly more accurate than the other build angles.
3. Artificial aging demonstrates a significant influence on trueness for SLA.
4. There are significantly larger positive deviations at all build angles after aging; SLA may have a higher fracture risk due to aggravating fit.
5. After aging, the magnitudes of positive deviations are also posterior for all printed resins and evenly distributed for those subtractively manufactured.

## Figures and Tables

**Table 1:** Showing Material composition of AM materials.

SLA (Dental LT Clear) [40]	7,7,9-trimethyl-4,13-dioxo-3,14-dioxa-5,12-diazahexadecane-1,16-diyl bismethacrylate (50–75%) 2-hydroxylethylmethacrylate (10–20%) Reaction mass of Bis(1,2,2,6,6-pentamethyl-4-piperidyl) sebacate and Methyl 1,2,2,6,6-pentamethyl-4-piperidylsebacate (<10%) Diphenyl(2,4,6-trimethylbenzoyl)phosphineoxide (1–5%) Acrylic acid, monoester with propane-1,2-diol (0.1–1%) Ethylenedimethacrylate (<10%) 2-hydroxyethylacrylate (0.1–1%) Mequinol,4-methoxyphenol,hydroquionemonomethyl ether (<0.1%)
DLP 1 (Luxaprint OrthoPlus) [41]	Dimethacrylate Resin (50–70%) EBPADMA (20–40%) Diphenyl(2,4,6-trimethylbenzoyl)phosphineoxide (1–2%)
DLP 2 (V-Print Splint) [42]	Polyesterdimethacrylate (50–100%) BIS-EMA (25–50%) Triethyleneglycoldimethacrylate (5–10%) Hydroxypropylmethacrylate (5–10%) Diphenyl(2,4,6-trimethylbenzoyl)phosphineoxide (≤2.5%) Butylatedhydroxytoluene (≤2.5%)

**Table 2:** Post-processing protocols for AM.

Printing Technique	Printer	Resin	Post Processing
SLA	Form 3	Dental LT Clear	Washing in Formwash (Formlabs) with 99% IPA for 20 min. Drying with compressed air. Post-curing in Form Cure (Formlabs) for 20 min at 80 °C. 3 min precleaning in IPA ultrasonic bath.
DLP 1	3Demax	LuxaPrint Ortho Plus	Rinsing in DMG 3Dewash with 99% IPA—Material-specific program. Drying with compressed air. Curing with DMG 3Decure: program specified for the material.
DLP 2	Solflex 170	V-Print Splint	2 min main cleaning in fresh IPA ultrasonic bath. Drying with compressed air. Post-exposure in Otoflash G171: 2000 flashes (10 per second), (NK-Optik GmbH, Baierbrunn, Germany). 2 min cooling, another sequence of 2000 flashes.

## Data Availability

Data are contained in the article.
